# What is the evidence for mirtazapine in treating cancer-related symptomatology? A systematic review

**DOI:** 10.1007/s00520-019-05229-7

**Published:** 2019-12-19

**Authors:** Guillaume Economos, Natasha Lovell, Anna Johnston, Irene J. Higginson

**Affiliations:** 1 Cicely Saunders Institut, Departement Palliative Care Policy and Rehabilitation, King’s College London, 10 Cutcombe Rd, Brixton, London, SE5 9PJ UK; 2 Hospices Civils de Lyon, Centre Hospitalier Lyon Sud, Palliative Care, 165 chemin du Grand Revoyet, 69310 Pierre-Bénite, France

**Keywords:** Mirtazapine, Neoplasms, Palliative care, Supportive care in cancer, Polysymptomatology

## Abstract

**Purpose:**

Cancer patients often experience multiple distressing symptoms which are challenging to manage. It would therefore be helpful to find a treatment that alleviates more than one symptom, to avoid polypharmacy: mirtazapine has been used in several studies for this purpose. The objective of this study was to assess the effectiveness and safety of mirtazapine in alleviating one or more frequently encountered cancer-related symptoms.

**Methods:**

Systematic review of clinical trials in English or French. Eight databases were searched. Included studies assessed the effectiveness of mirtazapine in alleviating one or more frequently encountered cancer-related symptoms. Comparator and validated assessment tools were required. Studies were independently appraised by two investigators before data synthesis.

**Results:**

The search yielded 1898 references, from which we identified 12 relevant articles evaluating highly heterogeneous outcomes. These were two randomised-controlled (RCTs), three non-randomised controlled, and seven non-randomised non-controlled trials. In total, 392 participants were included and 185 were in RCTs. No study assessed the effectiveness of mirtazapine in alleviating symptoms at the same time, but some considered more than one symptom. Overall, the data was of poor quality, limited by small sample size and bias. However, mirtazapine showed effectiveness in treating depression, anxiety, sleep disorders, emesis and neuropathic pain. Across all studies, mirtazapine is safe to use, with drowsiness and dizziness the most common side-effects.

**Conclusion:**

Study design and small sample sizes limit the ability to interpret results. Trials to assess the impact of mirtazapine or other medicines in alleviating multiple symptoms would be valuable.

**Electronic supplementary material:**

The online version of this article (10.1007/s00520-019-05229-7) contains supplementary material, which is available to authorized users.

## Introduction

Cancer patients often experience multiple distressing symptoms simultaneously [1]. The experience of multiple symptoms at the same time is referred to as polysymptomatology and requires multiple medications to mitigate their effect [2]. The most burdensome includes fatigue, pain, lack of energy, weakness and loss of appetite, affecting more than half of patients with advanced cancer [1, 3]. These symptoms are a challenge to assess and treat, and very few drugs are licenced for this purpose [4, 5]. In this frail and multimorbid population, polymedication increases the risk of drug interactions and side effects [6, 7]. One approach to tackle this is identifying a single medication which can effectively treat multiple symptoms.

Mirtazapine [8], a noradrenergic and specific serotonergic antidepressant, has proved effective in the treatment of depression in the cancer population [9]. It has also been evaluated in several studies to alleviate other cancer-related symptoms. This pre-synaptic α2adrenoreceptor antagonist increases the central noradrenergic and serotoninergic neurotransmission. Whilst the cause of its effectiveness as an antidepressant remains unclear, it is hypothesised to be due to a blockage of pre-synaptic α2 receptors leading to the release of norepinephrine, and a better availability of neurotransmitters in the synapse. It also antagonizes α2 heteroreceptors leading to an increment of serotonin release. Besides these central noradrenergic and serotoninergic effects, mirtazapine has an affinity to the anti-H1 receptor and is an 5-HT3 antagonist [10], which could be relevant in treating sleep disorders, appetite and breathlessness [11]. With this pharmacological profile, mirtazapine may be effective for the treatment of multiple symptoms, particularly those associated with cancer [5, 8, 12].

Mirtazapine is reported to be a safe antidepressant drug in the cancer population. It is almost completely metabolized by the liver and has a low-drug interaction risk, thus, allowing its use in advanced renal failure [10, 13]. However, some authors report drug-related symptoms such as dry-mouth, sedation, increased appetite and weight gain [14]. Sedation, increased appetite and weight gain are specific to mirtazapine, and could be useful in the cancer population who commonly experience poor sleep and a lack of appetite.

The effectiveness and safety of mirtazapine in alleviating multiple symptoms in cancer populations remain unclear. This review aims to address this question.

## Material and methods

We performed a systematic review of the literature using eight different databases to identify studies relevant to our research question.

The full protocol is available in supplementary material 1.

### Data sources

To identify relevant studies, we searched on Medline, Scopus, Web of Science, Central and EMBASE. We searched for grey literature on Clinical Trials, the WHO ICTRP and OpenGrey. Investigators were contacted by e-mail to request any unpublished study details identified using the clinical trial databases. Additional records were identified using related articles and references as well as by open searches. The inclusion time frame covered all databases until the 15th of January 2019.

Research algorithms were designed to fit with each database to improve the sensitivity of the search (supplementary material 2). The titles and abstracts (if available) of yielded records were screened for inclusion and exclusion criteria to evaluate their eligibility. Full text articles were then read and non-relevant articles excluded.

### Article selection

We included only primary literature: randomized controlled trials (RCTs), cohort studies, case-controlled and non-randomised experimental studies, reporting original studies written in English or French languages. Experimental studies were required to use a control.

Included studies concerned patients diagnosed with cancer, excluding cancer survivors, with one or more of the following symptoms: depression, anxiety, sleep disorders, nausea, anorexia, weight loss, breathlessness, pain, constipation, fatigue and drowsiness. These symptoms were chosen based on the fact that they are the most frequently encountered symptoms in cancer [3] and could potentially be addressed using mirtazapine given its pharmacological profile [8]. The primary outcome of studies was improvement of one of the listed symptoms.

### Data extraction

Data was extracted independently by two authors (GE, NL) regarding the effectiveness of mirtazapine (primary or secondary outcome) and the safety of its use. Authors extracted data on the year of publication and country of the study, number of participants, doses and modes of administration of mirtazapine and the comparator, follow-up completion rate, assessed symptoms and the tools used for assessment, results of the analysis, reported toxicity, adverse events and reasons for withdrawals.

### Data synthesis

Two authors (GE, NL) independently assessed the risk of bias and quality of studies using Cochrane Collaboration’s tools for RCTs and crossover studies, and the checklist for non-randomised experimental studies provided by the Johanna Briggs Institute for non-randomised experimental studies (Table 1).

**Table 1:**
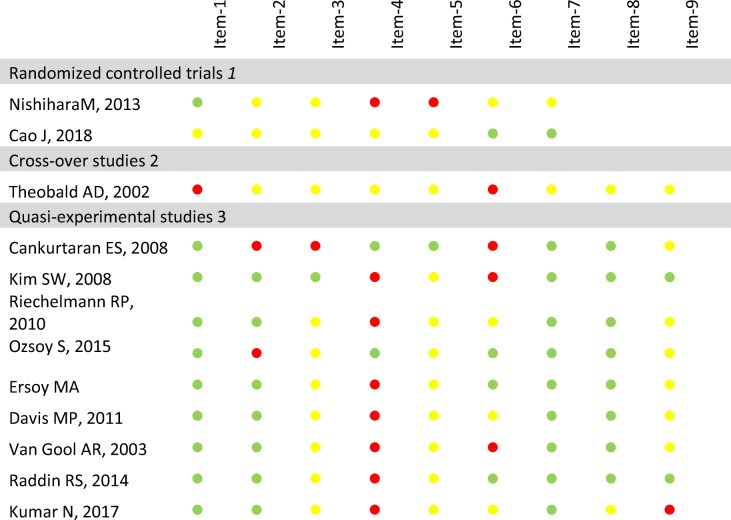
Assessment of the studies’ risk of bias according to their designs

^1^Randomized controlled trials’ risk of bias assessed using the Cochrane collaboration tool for assessing risk of bias:  low risk of bias,  unclear risk of bias,  high risk of bias

*Item 1* random sequence generation, *Item 2* allocation concealment, *Item 3* blinding participant and personnel, *Item 4* blinding outcome assessment, *Item 5* incomplete outcome data, *Item 6* selective reporting, *Item 7* other source of bias, *Items 8* and *Item 9* are not suitable for randomized controlled trials

^2^Cross-over trials’ risk of bias assessed using the Cochrane collaboration tool for assessing risk of bias:  low risk of bias, \ unclear risk of bias,  high risk of bias

*Item 1* appropriate crossover design, *Item2* randomized treatment order, *Item 3* carry-over effect, *Item 4* unbiased data, *Item 5* allocation concealment, *Item 6* blinding, *Item 7* incomplete outcome data, *Item 8* selective outcome reporting, *Item 9* other bias

^3^Quasi-experimental studies’ risk of bias assessed using the Joanna Briggs Institute Checklist:  adequate,  unclear,  inadequate

*Item 1* causes and effects are clearly defined, *Item 2* similarity in participants, *Item 3* similarity in treatments, *Item 4* existing control group, *Item 5* multiple measurement, *Item 6* follow-up completion, *Item 7* outcome measurement comparable, *Item 8* outcome measurement reliable, *Item 9* appropriate statistics

Data were summarized according to the level of evidence permitted within the study design (Table 2, supplementary material 3) and the risk of bias for each study (Table 1). Evidence for each symptom was assessed following the GRADE practice recommendations [15]. If the authors disagreed on data, an external opinion was sought.

**Table 2:** Summary of the main findings

	GRADE’s quality of evidence	Symptom	Data summary
Targeted symptoms	Low	Nausea and vomiting	6 studies (236 patients) of which 1 RCT (95 patients) ☞ Could mitigate chemotherapy-induced emesis within 3 days of treatment in addition to other anti-emetic drugs. Has not been proven to mitigate radiotherapy induced emesis. No evidence available in other situations.
	Very low	Pain	4 studies (140 patients), of which 1 RCT (25 patients) ☞ Was more effective to treat neuropathic pain from day 14 than pregabalin alone.
		Depression	8 studies (249 patients) ☞ Could be effective earlier than with compared antidepressants.
		Anxiety	6 studies (214 patients) ☞ Could improve anxiety, could be effective from day 15.
		Sleep disorders	5 studies (155 patients) ☞ Could improve every stage of sleep, and extend the length of sleep. Could be efficient from week 1.
		Anorexia	3 studies (113 patients) ☞ Weak evidence in effectiveness of improving appetite.
		Loss of weight	4 studies (148 patients) ☞ Weak evidence in the effectiveness of weight gain.
	Not applicable	Breathlessness	1 study (17 patients) ☞ Studies are underpowered to make a statement.
Side effects	Very low	Drowsiness and fatigue	2 studies (35 patients). ☞ The studies did not report any changes in drowsiness and fatigue, however, these two are often reported as side effects.
	Not applicable	Dizziness	No study available; however, dizziness is often reported as a side effect.
		Constipation	No study available in this specific population, but a well-known side effect in the general population.

Regarding the predictably high heterogeneity of studies, no meta-analysis has been planned.

## Results

### Search results

**Fig. 1:**
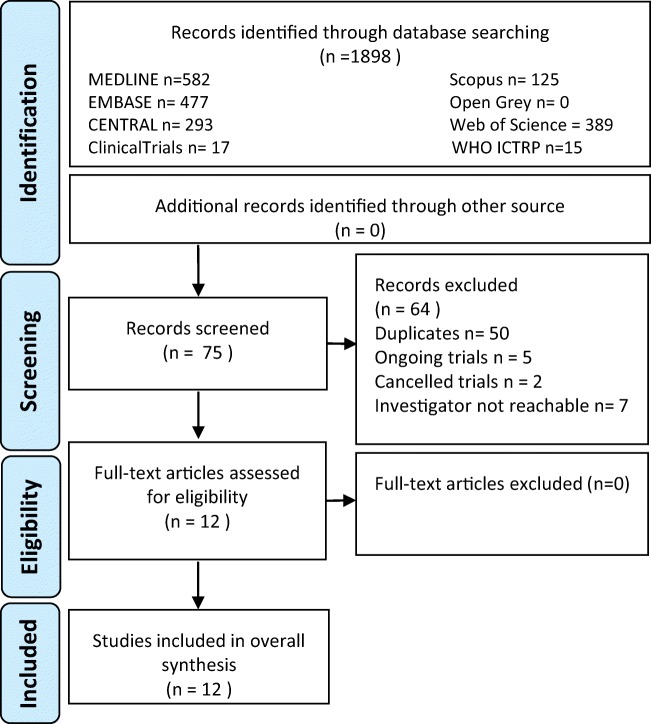
Flow-chart (based on the CONSORT statement)

The electronic search yielded 1898 references overall, 582 from Medline, 477 from EMBASE, 293 from Central, 389 from Web of Science, 125 from Scopus, none from OpenGrey, 17 from Clinical Trials and 15 from the ICTRP. After this screening, 75 articles were identified as relevant. Of these, 50 were duplicates. From studies identified using clinical trials registries, five trials were ongoing, two had discontinued, six investigators did not answer our requests and one informed us that the study was currently under submission process. After this, 12 relevant studies remained with no additional records identified (Fig. 1). Three studies were presented as RCTs, including 53 [16], 25 [17] and 95 patients [18]. However, closer scrutiny of the designs revealed that, in one, the control group was made of people refusing to take antidepressant medications [16]. Therefore, this study has been considered a non-randomised experimental study for the purpose of this review.

One was a crossover trial [19], and nine were non-randomised experimental studies [5, 16, 20–25]. The longest duration of treatment was 6 months [20], and the shortest was 3 days [18]. All articles were in English.

Overall, the evidence was highly heterogeneous (Table 1 and supplementary material 3) and the quality of the studies’ reported was poor, with important concerns about the risk of bias (Table 2).

### Effectiveness of mirtazapine in cancer-related symptoms

#### Evidence from randomised-controlled trials

Two studies used randomised-controlled designs, although mirtazapine was not compared with a placebo in either. The studies assessed the effectiveness of mirtazapine on two different symptoms: emesis [18] and pain [17].

Cao et al.’s study aimed to assess the effectiveness of mirtazapine in addition to usual anti-emetic therapies in the treatment of chemotherapy-induced emesis [18]. The study included 95 breast cancer patients undergoing cisplatin chemotherapy. The intervention group received mirtazapine in addition to aprepitan, a 5HT3 receptor antagonist and dexamethasone 7.5 mg. The control group received the same medications except mirtazapine. Response was assessed as “complete response to vomiting” (no emesis and no rescue treatments) and “complete control” (defined as no emesis, no rescue treatment and no more than grade 1 nausea). In the first cycle, delayed and overall complete response rates were significantly higher with mirtazapine (78.3 versus 49% *p* = 0.003, and 58.7 versus 34.7% *p* = 0.019). Similar results were observed in the 3rd cycle. The study closed early due to slow enrolment, and the interpretation of results is limited by a small sample size.

Nishihara et al.’s study compared antidepressant drugs used as adjuvants with pregabalin and opioids for intractable painful bone metastases in mixed cancer types [17]. The authors compared pregabalin 50 mg three times daily, pregabalin 25 mg three times daily combined with mirtazapine 7.5 mg twice daily and pregabalin 25 mg three times daily combined with imipramine 5 mg twice daily. Authors also recorded the average use of opioids in the three arms. The trial included 25 patients treated for 15 days; a numerical rating scale was used to evaluate average intensity of pain and intensity of paroxysmal pain over the past 24 h. The results found a clinically important difference over 2 [26, 27] in the total pain score intensity and in the paroxysmal pain intensity from the 1st day of use in all 3 arms of the trial. This decrease was significantly higher in the arm with mirtazapine and imipramine than in the arm with pregabalin only, and results were higher in the mirtazapine arm than the imipramine arm during the first day of treatment. There was no difference in the daily opioid dose for any of the arms.

#### Evidence from non-randomised controlled trials

Three non-randomised controlled trials were identified [16, 23, 24]. Two compared mirtazapine with other antidepressant drugs, one with imipramine and a control group [16], and the second with citalopram [24]. The last one compared mirtazapine with a non-interventional control group. The studies attempted to assess a wide range of symptoms including depression, anxiety, pain, appetite, emesis, insomnia, weight loss and fatigue using validated tools. Overall, the sample sizes were small (43.7 participants on average) with a high risk of bias (Table 1).

Cankurtaran et al. report a randomised-controlled trial; however, in this study, the control group was participants who had refused the intervention (mirtazapine) [16]. We have therefore considered this to be a non-randomised controlled trial. The study included 53 patients over a 6-week period with a follow-up completion rate of 0.66. Participants were cancer patients with various diagnoses. One arm received an unspecified dose of mirtazapine in addition to supportive therapy for 6 weeks, the second one received imipramine in addition to supportive therapy and the third (who had refused antidepressant treatment) received only supportive therapy. The evaluated outcomes were nausea, vomiting, reduced appetite, weight, sleep disorders, depression, anxiety and pain. Results did not show any difference in nausea or vomiting (using a single symptom scale). When assessing for pain, no difference was found between arms using a numerical rating scale. Anxiety and depression were assessed using a validated tool in cancer, the hospital anxiety and depression scale (HADS) [28, 29]. The study found a statistically and clinically significant difference [30] in anxiety (− 3.7 points, *p* = 0.025) and depression (− 4.7 points, *p* = 0.003) for patients taking mirtazapine, compared with imipramine and control. The effectiveness on sleep disorders was assessed using the Hamilton depression rating scale (HAM-D) which is validated in cancer [31]. For initial, middle and late insomnia, only the mirtazapine group showed improvement (*p* = 0.001, *p* = 0.001, *p* = 0.003). Using single symptom scales, no significant difference was found for appetite or weight change in the mirtazapine group when compared with the other arms.

Raddin et al. report the results of a non-randomised controlled study including 18 patients over a 9-week period [24]. The follow-up completion rate was 0.86. Participants received mirtazapine (starting dose 7.5 mg, escalated to 15 and then 30 mg as appropriate) or citalopram (starting dose 10 mg, escalated to 20 and then 40 mg as appropriate). Allocation was not concealed and was decided by clinical experience. The authors assessed depression using the Patient Health Questionnaire 9 (PHQ-9) at baseline, weeks 1, 2, 3, 4, 6 and 9. In this study, depression did not significantly improve in the overall cancer sample when evaluated using the PHQ-9. However, a sub-analysis which excluded actively dying patients showed a significant and clinically important difference [32] of 7.6 (95% CI = [2.9–12.2]) after 9 weeks of treatment. The quality of sleep was evaluated using the Pittsburgh Sleep Quality Index (PSQI) [33]. The study reports a non-significant improvement in sleep quality when assessed using the PSQI score (11.0 versus 8.6, 95% CI = [− 2.2–6.9]) and a non-significant improvement of the hours of sleep (5.9 versus 7.5, 95% CI = [− 0.3–3.5]). The study did not find any significant difference for weight or fatigue across the different arms.

Oszoy et al. report an open-labelled study assessing the outcomes of radiotherapy-induced cachexia treated with mirtazapine 15-30 mg for 6 months in patients with head and neck cancer [23]. The interventional group was made of patients diagnosed with major depression using the Hamilton depression rating scale, and they were compared with a control group who did not have a diagnosis of cancer or depression. The primary outcome of this study was to assess the effectiveness of mirtazapine on the level of two hormones involved in the regulation of food intake (ghrelin and leptin); secondary outcomes were assessment of weight and body mass index. The results are challenging to analyse, and no conclusion can be reached as baseline characteristics highly differ between the groups.

#### Evidence from non-randomised non-controlled trials

We recorded seven non-randomised non-controlled trials which were all before and after designs [19–22, 25, 34, 35]. They assessed a number of symptoms including the following: depression, anxiety, emesis, insomnia, anorexia, weight loss, breathlessness, fatigue and pain. The studies had small sample sizes (on average 24.1 participants in each study) and a high risk of bias.

Theobald et al. report a 6-week open-label crossover trial comparing the effectiveness of mirtazapine 15 mg versus mirtazapine 30 mg in cancer patients experiencing pain [19]. Evaluated outcomes were pain, depression, nausea and appetite. The study included 20 patients over a 6-week period with a low follow-up completion rate (0.55). The authors assessed pain, appetite, and nausea and vomiting using a numeric rating scale and found no change for any symptom between baseline and end-point. However, patients did report feeling less concerned about their weight at week 4 (F = 12.9, *p* < 0.01) and week 7 (F = 4.7, *p* < 0.05) when compared with baseline. Depression was assessed using the Zung self-rating depression scale (ZSDS) which is validated for cancer [36]. The authors report a significant improvement in the ZSDS scores at week 7 (F = 8.2, *p* < 0.05).

Ersoy et al. report a before-after trial which followed up 19 patients treated with mirtazapine 15 mg daily for 6 months [20]. The study reports a clinically significant improvement in depression using the 17-item Hamilton rating scale with a drop from 21.4 ± 4.9 at baseline to 6.5 ± 3.2 at end-point (*p* < 0.001) [37, 38]. This improvement was significant for each sub-index of the scale rating anxiety, depression and the quality of sleep.

Riechelmann et al. report a before-after trial which followed up 21 participants for 8 weeks of treatment with mirtazapine 15-30 mg daily [21]. The primary outcome was a gain of at least 1 kg after 4 weeks of treatments and secondary outcomes were appetite and quality of life. At week 4, on intention to treat, 24% of participants had gained 1 or more kilogrammes with a median gain of 1.5 kg (ranging from 1 to 3.6); all respondents reported an improvement in their appetite (of more than 2 points) on the Edmonton Symptom Assessment Scale.

Kim et al. describe the results from a before-after trial which followed up 39 participants treated with mirtazapine 15 mg daily for 4 weeks [22]. The primary outcomes were the Chonnam National University Hospital Leeds Sleep Evaluation Questionnaire (C-LESQ) for the quality of sleep, and the Clinical Global Impression (CGI) scale for nausea. The amount of sleep increased from 3.6 at baseline to 6.8 h per day at end-point (*p* < 0.001), the ease of getting sleep improved from 4.2 to 2.4 (*p* < 0.001), the quality of sleep improved from 4.3 to 2.6 (*p* < 0.001) and the ease of waking in the morning improved from 3.2 to 2.5 (*p* < 0.001). In the sub-population of patients experiencing nausea at baseline (*n* = 28), the rating of nausea decreased from 4.6 ± 1.3 at baseline to 2.6 ± 1.9 at the end-point (*p* < 0.001).

Kumar et al. present the descriptive results for a before-after trial including 30 patients treated with mirtazapine 7.5 mg daily for 15 days [34]. Anorexia was a secondary outcome reported using a single symptom scale. At baseline 10.3% of participants experienced mild anorexia, 41.4% moderate anorexia and 62.1% severe anorexia. At end-point, 23.3% did not experienced anorexia anymore, 62.1% experienced mild anorexia, 13.8% moderate anorexia, and none experienced severe anorexia.

Mellar et al. report a before-after trial including 57 patients treated with mirtazapine 15 mg daily (increased to 30 mg daily after 1 week) for 15 days. They assessed insomnia, nausea and anxiety using the EORTC QLQ-C30 sub-scales and considered a response if the difference was over 1 point on the sub-scale. In intention to treat, insomnia and anxiety had a response rate of 33%.

#### Safety of mirtazapine’s use in cancer populations

Only two studies included a validated tool to evaluate side effects or the toxicity of mirtazapine in their design [22, 35]. Additionally, one study reported outcomes about fatigue and drowsiness using a validated scale [21] and one about the clinical global impression [25].

One open-labelled study including 42 participants with a follow-up completion rate of 0.4 used the UKU side effect rating scale [22] which has been developed to assess and rate the side effects of psychotropic treatments [39]. It has not however been validated in cancer. In this study, authors report that sleepiness/sedation was experienced after introduction in 36% of subjects. However, sleepiness/sedation appeared to decrease over the time, 19% of patients experienced  increased sedation after the seven first days of treatment but they were only 8% after 14 days and none continued to experience an increased sedation on day 28. When compared with baseline; at day 7, 19% had a worst sleepiness/sedation, they were and 8% on day 14 and 0% on day 28.

Additionally, 48% of patients already had sleepiness/sedation before the medication. Sixty percent of those patients improved sleepiness when compared with baseline.

A non-randomised experimental study used the Common Terminology Criteria for Adverse Events to report adverse effects during the study period [35]. This tool has been developed to assess the side effects of treatments in cancer populations [40]. In this study, the author reports 4 patients experiencing grade 3 toxicity in the first week, 4 with a grade 3 toxicity in the second week and 1 with a grade 4 toxicity in the second week.

An open-labelled study including 17 participants evaluated fatigue and drowsiness using the ESAS subscales [21]. Whilst it did not find any difference in drowsiness, the ESAS fatigue subscale had a median decrease of 3.5 points, corresponding to a clinically important difference.

Overall, among all patients receiving mirtazapine and for whom the studies report the number of side effects (*n* = 192) [19–22, 24, 35], the most frequent side-effect was the somnolence/drowsiness experienced by 48% of patients (*n* = 25). This concurs with the comments made in several studies reporting that sedation was the most important side-effect, responsible for the largest amount of withdrawals [18, 25]. The second most frequent side effect was dizziness which occurred in 13.4% (*n* = 7) of the participants. The next was fatigue, experienced by 9.6% (*n* = 5) of the patients, which was also supported by several comments found in the studies [17, 24]. After these symptoms, by order of frequency, patients reported delirium and xerostomia, weight gain, nausea, intentional tremor, restless legs, insomnia and blurred vision. Among all studies, the withdrawals were mostly reported to be unrelated to adverse events.

Overall, only a few patients treated with mirtazapine had side effects important enough to withdraw from a study. The most frequent side effects were somnolence/drowsiness, dizziness and fatigue.

## Discussion

The studies presented in this review provide low level evidence for treating polysymptomatology, limited by sample size with a high risk of bias. It is therefore not possible to recommend the use of mirtazapine for multiple palliation. However, the results confirm the effectiveness of mirtazapine in psychiatric symptoms like depression and anxiety. They are also encouraging for its effectiveness in several other symptoms, in particular, the treatment of sleep disorders, pain and cancer-related emesis. These findings should inform future RCTs to better determine the effectiveness of mirtazapine in these symptoms.

Moreover, European populations are ageing and the problem of polypharmacy is now a main concern of geriatricians [41, 42]. With an ageing population, and cancer incidence increasing with age, we can expect a rise in the number of advanced cancer patients and palliative patients undergoing polypharmacy treatments. This represents a potential risk for safety as well as for the quality of life of these patients. A key to improving the management of ageing cancer populations would be to evaluate medications that decrease the risks related to polypharmacy whilst simultaneously improving quality of life and multiple symptoms [43]. Therefore, future RCTs should aim to determine the effectiveness of alleviating multiple symptoms and quality of life.

Whilst this review did not aim to assess the effectiveness of mirtazapine in improving quality of life, four studies evaluated this as a secondary outcome, and overall, they suggested an improvement in quality of life for patients taking mirtazapine [19, 21, 24, 35]. In addition, Van Gool et al.’s paper found an increase in the clinical global impression scale, which measures the perceived efficacy of the medication in improving the global clinical state of the patient. This improvement is suggestive of a treatment response and improvement in symptom severity. Global clinical improvement might also reflect an improvement in quality of life. It supports the importance of assessing the potential improvement in quality of life whilst using this medication to alleviate multiple symptoms.

Our findings suggest that mirtazapine could be of interest in alleviating symptoms strongly associated with depressive disorders, such as anxiety and sleep disorders [44]. The population of cancer patients is at high risk of psychiatric and sleep disorders [45, 46], and the use of mirtazapine to alleviate more than one symptom could be a good alternative to multiple medications. However, effectiveness in treating these three symptoms might be explained through their categorisation as part of the same cluster of symptoms [47]. Therefore, experiencing one of these symptoms can have a worsening impact on the others [48]. For this reason, the effectiveness of mirtazapine in treating anxiety and sleep disorders could be an indirect consequence of a direct action on depressive disorders.

Regarding pain management, Nishihara’s study results is informative for future research [17]. Despite a high risk of bias, the significant changes might reflect a benefit from mirtazapine in treating neuropathic pain. This effect on neuropathic pain could be of great interest in the cancer population. This population often experiences neuropathic pain, either because of a direct effect of the neoplasm or side effects of the treatments [49]. Moreover, chronic pain and especially chronic neuropathic pain are common risk factors for depressive disorders [50], and some authors suggest that, considering that they are part of the same symptom cluster, an improvement in neuropathic pain may lead to an improvement in sleep quality [51]. Therefore, the effectiveness of mirtazapine in chronic neuropathic pain management could be of interest in more ways than one by treating the underlying symptom cluster of pain-depression-sleep disorders. Serotonin noradrenaline reuptake inhibitors (SNRIs) are antidepressants approved to treat neuropathic pain. Their action on neuropathic pain is not fully understood; however, it might be mediated by enhancing serotonin and noradrenaline in the spinal and supraspinal structures [52]. Besides, tricyclic antidepressants are also approved in this indication. Like mirtazapine, tricyclic antidepressants inhibit serotonin and noradrenaline reuptake in the synapse, resulting in a central noradrenergic and serotoninergic neurotransmission increase [53]. These shared pharmacological pathways between mirtazapine and other medications licenced for treating neuropathic pain could explain the potential effectiveness of mirtazapine for this indication.

Mirtazapine may also be an interesting antidepressant to treat multiple symptoms because of its effects on appetite and weight [14]. Mirtazapine’s side effects might be of great interest, particularly because malnutrition is a cause of treatment intolerance and shortens the life expectancy of advanced cancer patients [54]. For these reasons, mirtazapine may be the preferred option when treating depression in cancer patients. To date, evidence for the use of mirtazapine to improve weight gain and appetite is lacking but studies are currently ongoing to address this.

Another interesting symptom for which no treatment is licensed in Europe is breathlessness. Evidence is lacking to support the use of mirtazapine in alleviating breathlessness; however, some pilot studies have shown encouraging results in alleviating breathlessness in advanced lung disease conditions, including lung cancers [55]. Mirtazapine appears to be a promising candidate to pursue, but definitive randomized controlled trials are required to determine its efficacy and safety in this setting.

## Limitations

Our review has several limitations. Whilst including grey literature, we cannot be certain that we have identified all studies. Some studies were excluded from the review because the data was not available. Publication bias is a common concern in interventional studies, especially in populations with life-threatening diseases, as many studies do not recruit or retain enough patients to have strong results, limiting their publication in peer-reviewed journals. Therefore, this review may have been impacted by publication bias. Additionally, we excluded studies that did not focus only on cancer patients. This decision was supported by the fact that most of these studies had “cancer-affected patients” as exclusion criteria. However, this choice potentially led to the neglect of relevant data.

## Conclusion

Overall, there are limited studies which aim to assess the effectiveness of mirtazapine in alleviating multiple symptoms in the cancer population and no studies which assess the use of mirtazapine to treat polysymptomatology. The study designs are mostly too weak to support strong results and often only include a small sample size. However, these results should inform further large RCTs which are able to determine the effectiveness of mirtazapine in treating multiple symptoms in the cancer population.

## Electronic supplementary material

ESM 1 (DOCX 51 kb)

ESM 2 (DOCX 13 kb)

ESM 3 (DOCX 21 kb)

## Data Availability

The datasets analysed during the current study are available from the corresponding author on reasonable request.
