# Supplementary material for: What is the evidence for mirtazapine in treating cancer-related symptomatology? A systematic review
Source: Support Care Cancer. 2019 Dec 19;28(4):1597–606. doi: 10.1007/s00520-019-05229-7 (PMC7036072; doi:10.1007/s00520-019-05229-7)
Supplement: Supplementary file 1 — (DOCX 51 kb) [file 520_2019_5229_MOESM1_ESM.docx]

**Effectiveness of the MIRTAZAPINE in advanced cancer polysymptomatology, what are the evidences? A systematic review.**

**VERSION 5**

**Investigators:** Dr Guillaume ECONOMOS, MD, MSc

Cicely Saunders Institute – King’s College London

10 Cutcombe Rd, Brixton, London SE5 9RJ

Guillaume.economos@kcl.ac.uk

T: 0033 6 6805 7146

Dr Natasha LOVEL, MBChB BSc MRCP

Cicely Saunders Institute – King’s College London

10 Cutcombe Rd, Brixton, London SE5 9RJ

Natasha.lovell@kcl.ac.uk

**Supervisor:** Pr HIGGINSON Irene J

Cicely Saunders Institute – King’s College London

10 Cutcombe Rd, Brixton, London SE5 9RJ

Irene.higginson@kcl.ac.uk

**Corresponding author:**

Dr Natasha LOVEL, MBChB BSc MRCP

Cicely Saunders Institute – King’s College London

10 Cutcombe Rd, Brixton, London SE5 9RJ

Natasha.lovell@kcl.ac.uk

**ABSTRACT**

**Context:**

MIRTAZAPINE is an antidepressant drug approved for use for the treatment of major depression.

This pre-synaptic alpha2adrenoreceptor antagonist increase central noradrenergic and serotoninergic neurotransmission.

It action on depression could be explained by blocking the presynaptic alpha2receptors leading to realizing norepinephrine and enhancing the availability of neurotransmitters in the synapse (or to an increase concentration of noradrenaline and an inhibition in serotonin releasing). It also antagonizes alpha2heteroreceptors leading to an increment of serotonin release. This action has central agonist effect on the serotonin and adrenergic systems.

It is almost totally metabolized in the liver. Allowing its use in advanced renal failure.

Most frequently encountered adverse effects are dry mouth, sedation and increase of appetite and body weight. MIRTAZAPINE has low drug-interaction risks its global safety profile allows its use in safety conditions in most of the cases.

Through its pharmacological profile, the MIRTAZAPINE has been proposed to treat multiple symptoms and there is an increasing interest especially for those associated with cancer.

Considering its supposed action on a wide range of symptoms, the MIRTAZAPINE might have a potential beneficial balance and its use could be relevant in advanced cancer situations to treat polysymtomatology.

All in all, MIRTAZAPINE is a promising treatment to treat multiple symptoms in palliative cancer situations, however, it remains gaps and discrepancies in the evidences about its effectiveness.

This review aims to assess evidences about effectiveness of MIRTAZAPINE in alleviating distressing symptoms in advanced cancer patients.

**Material and methods:**

We will conduct a systematic review from April 1982 to January 2019 in the following databases: MEDLINE, EMBASE, CENTRAL and GOOGLE SCHOLAR. The grey literature will also be explored.

We will search for meta-analysis, systematic reviews, randomized controlled trials, quasi-experimental studies and non-experimental studies which aims to assess the effectiveness of the MIRTAZAPINE in commonly encountered symptoms in advanced cancers patients. Symptoms of interest will be the pain, fatigue, nausea, anxiety, drowsiness, breathlessness, appetite, constipation and sleeping quality. Studies will have to use validated tools or numeric-scales to assess the symptoms and to compare the interventional group with a control. Studies will have to focus on patients with cancer with a minimum range of 50% of advanced cancer patients.

**Analysis:**

Analysis of the quality of studies will be made using standard recommended tools regards to their design.

Synthesis of data will be realized for each symptom separately (grouped in cluster in need) considering at first meta-analysis, systematic reviews and randomized control trials results. There results will be summarized using forest-plots.

If there are no such studies, other study’s results will be described.

**Key words:** MIRTAZAPINE, advanced cancer, symptom management

**TABLE OF CONTENTS:**

**Investigator list**

**Abstract**

**I- Background**

**II- Objectives**

2.1- Main Objective

2.2- Review question

**III- Review protocol**

3.1 - Searches

3.2 – Research algorythm

3.3 – Type of studies to be included

3.4 – Population of the study

3.5 – Intervention evaluated

3.6 – Outcomes evaluated

**IV- Data extraction**

4.1 – Selection process

**V- Strategy for data synthesis**

5.1 - Quality assessment for meta-analysis and systematic reviews

5.2 – Quality assessment for randomized controlled trials

5.3 – Quality assessment for quasi-experimental studies

5.4 – Quality assessment for non-experimental studies

5.5 – Global strategy for synthesis

**V- Study schedule**

**VII- References**

1. **Background:**

MIRTAZAPINE is an antidepressant drug approved for use for the treatment of major depression (1).

This pre-synaptic alpha2adrenoreceptor antagonist increase central noradrenergic and serotoninergic neurotransmission. Its action on depression could be explained by blocking the presynaptic alpha2receptors leading to realizing norepinephrine and enhancing the availability of neurotransmitters in the synapse. It also antagonizes alpha2heteroreceptors leading to an increment of serotonin release. This action has a central agonist effect on the serotonin and adrenergic systems.

It is almost totally metabolized in the liver. Allowing its use in advanced renal failure (2).

Most frequently encountered adverse effects are dry mouth, sedation, and increase of appetite and body weightv (3). These two last side effects are specific to the MIRTAZAPINE whereas other antidepressant drugs profiles are more likely to provide loss of weight and anorexia. MIRTAZAPINE has low drug-interaction risks and its global safety profile allows its use in safety conditions in most of the cases (4, 5).

Beside the central noradrenergic 1and serotoninergic effects, MIRTAZAPINE has an affinity to the histamine-H1 receptor and is a 5-HT3 antagonist (2). Through its pharmacological profile, the MIRTAZAPINE has been proposed to treat multiple symptoms and there is an increasing interest especially for those associated with cancer (6, 7).

Indeed, in most of the case, advanced cancer patient experience many symptoms at the same time and scales have been proposed to assess these symptoms in a same tool (8). Currently, patients are treated by various medications to treat these multiple symptoms. This use of multiple drugs might increase the like hood of pharmacological interactions and adverse effects. Therefore it could be relevant to evaluate the effectiveness of a unique molecule, such as MIRTAZAPINE, to treat multiple symptom and by that way, limiting the use of multiple drugs (9).

For instance, MIRTAZAPINE’s 5-HT3 antagonist action has been shown to be efficient to manage sleep disorders (10) or increase appetite (11). It has also been shown to be efficient to treat anxiety or hot flushes (6). However, information focusing on advanced cancer populations are lacking and

in a surprizing way, a recent letter reported a lower tolerance in advanced cancer patients than in the historically-treated population of depressive patients (12).

Considering its supposed action on a wide range of symptoms, the MIRTAZAPINE could be relevant in advanced cancer situations to treat polysymptomatic patients if its adverse effects are not overwhelming its benefits.

1. **Objectives:**

**2.1 - Main objective:**

The main objective of the review is to assess evidences about the effectiveness of MIRTAZAPINE to treat the cancer related symtpoms.

**2.2 - Review question:**

| Population | Intervention | Comparison | Outcome |
| --- | --- | --- | --- |
| Cancer-patients suffering from:   - Pain - Fatigue - Nausea - Anxiety - Drowsiness - Breathlessness - Appetite - Constipation - Bad sleeping quality | MIRTAZAPINE | NC | Modification in symptoms’ assessment (change in symptom)  Evaluation of side effects |

- Does the use of MIRTAZAPINE could be relevant to manage multiple discomfort symptoms in cancer situations?

1. **Review protocol:**

Methodological references: *Cochrane Handbook for Systematic Reviews of Interventions Version5.1* and *PRISMA-P 2015 Statement.*

- 1. **Searches:**

1. **Electronical searches :**

**Amended after reviewers’ comments:**

“The following databases will be explored from April 1982 to January 2019: MEDLINE (Pubmed), EMBASE, Central, Scopus, Web of science.

These databases have been chosen because it has been described as the optimal searching strategy (13).

We will start our inclusion period on April 1982 as the first article about the MIRTAZAPINE has been published on this date.

To identify unpublished or ongoing studies, we will use the following databases: OpenGrey, CLINICAL TRIAL, and The WHO Trial Portal (ICTRP).”

1. **Other searches :**

If a study is identified as possibly relevant for the review but is not published yet (on CLINICAL TRIALS for instance), investigators will be contacted. They will be kindly asked to provide either the unpublished article or the study protocol and their results.

**3.2- Research algorithm:**

**Amended after reviewers’ comments:**

“For our searches on MEDLINE, as MEDLINE uses MeSH words to index articles, we will use the following algorithm of research that uses relevant MeSH words as well as free-text research terms:

((((mirtazapine) OR ("Antidepressive agents"[Mesh]))) AND (("Neoplasms"[Mesh]) OR ("Palliative Care"[Mesh]) OR ("Hospice Care"[Mesh]))) AND (("nausea"[Mesh]) OR ("weight loss"[Mesh]) OR ("fatigue"[Mesh]) OR (“constipation”[Mesh]) OR ("pain"[Mesh]) OR ("Pain management"[Mesh]) OR ("dyspnea"[Mesh]) OR ("anorexia"[Mesh]) OR ("sleep initiation and maintenance disorders"[Mesh]) OR ("sleep wake disorders"[Mesh]) OR ("anxiety"[Mesh]) OR (“depression”[Mesh])”

Other databases does not use MeSH words for referencing the articles. They search in the whole article the words used for the research algorithm. Therefore we only will use one single synonym for each assessed symptom in these databases.

- 1. **Type of studies to be included:**

1. We will include all published or unpublished studies.
2. Only primary literature articles will be included, considering only randomized controlled trials, cohort studies (prospective or retrospective), case-controlled and quasi-experimental studies. Results of previous trials might be reported in full articles, brief reports or letters.

We will exclude all secondary analysis (such as Meta-analysis, systematic reviews and narrative reviews), clinical case studies (and case report), perspectives, opinion, commentaries, editorials, guidelines, news articles, conference abstracts and all the trade publication articles.

1. The experimental studies must use a control group compared to the use of the MIRTAZAPINE.

Controls can be pre-intervention, placebo or any other medication or intervention.

1. The studies have to focus only on patients with cancer.
2. Studies will have to aim to evaluate the effectiveness of MIRTAZAPINE in alleviating on one or more of the following symptoms:

- Pain
- Fatigue
- Nausea
- Anxiety
- Drowsiness
- Breathlessness
- Appetite
- Constipation
- Sleeping quality
- Depression

1. In English or French language.
2. Studying humans
   1. **Population of the study:**

The population must include:

- Patients over 18 years old.
- Males or females.
- In-patients or out-patients.
- A specific type of cancer or not.
- Treated using MIRTAZAPINE.

NB: receiving a cancer specific therapy (such as chemotherapy, radiotherapy or immunotherapy…) is not a criteria of exclusion.

We will exclude cancer survivors.

- 1. **Intervention evaluated:**

MIRTAZAPINE’s use.

Every MIRTAZAPINE galenic forms, way of introduction, duration of treatments and doses will be considered.

For Trials, all types of comparator groups will be included whether they are a non-exposed control group (including pre-intervention in quasi-experimental designs) or a group exposed to another intervention.

- 1. **Outcomes evaluated**

We will include the studies using the followings to assess the severity of a symptom:

- A validated scale or tool

Or

- A numeric-scale (for instance, a Likert scale)

Or

- A single-symptom assessment scale

The symptoms of interest will be the most commonly encountered symptoms in cancer-patients (1) on which mirtazapine might have an impact considering its pharmacological profile .

Therefore, the following symptoms will be assessed:

- Pain
- Fatigue
- Nausea
- Anxiety
- Drowsiness
- Breathlessness
- Appetite
- Constipation
- Sleeping quality
- Depression

1. **Data extraction :**
   1. **Selection process:**

The investigator will screen titles and abstracts of the articles identified using the algorithm of research.

He will select the articles which meet the following inclusion criteria: studies that evaluate the use of MIRTAZAPINE in cancer patients only, compared to a control (except for meta-analysis and systematic reviews) to treat one of the evaluated symptoms (pain, fatigue, nausea, anxiety, breathlessness, appetite, constipation and sleeping disorders).

The full article will be read and analysed by the investigator. 25 % of them will be double-checked. If the article still responds to all inclusion criteria, it will be included in the review.

If the article is finally excluded, the reason for this exclusion will be recorded.

1. **Strategy for data synthesis :**

Different quality assessment tools will be uses depending on the type of the study.

For each included study, we will record the main objective, the design, the symptom evaluated, the population included, the intervention and the control group, the main outcomes and any relevant remark that could underling a specific aspect of the study.

- 1. **Quality assessment for randomized controlled trial:**

To assess the quality of randomized controlled trials, we will use the Cochrane Collaboration’s Tool for Assessing Risk of Bias for Randomized Controlled Trials (Higgins J, Green S. Cochrane Handbook for Systematic Reviews of Interventions Version 5.1.0. 2011. [updated March 2011]. Available from www​.cochrane-handbook.org. )

- 1. **Quality assessment for quasi-experimental studies:**

For assessing the quality of quasi-experimental studies, we will use the Johanna Brigs Institute critical appraisal Checklist for quasi-experimental studies.

- 1. **Quality assessment for non-experimental studies (cohort studies):**

We will use the Newcastle-Ottawa scale to assess the quality of non-randomized included studies.

- 1. **Global strategy for synthesis:**

Studies will be grouped by symptom of interest.

To evaluate the effect of the MIRTAZAPINE in the management of a symptom, we will use randomized controlled trials.

For every symptom evaluated, if a study assesses any modification in this symptom as a side effect of the treatment, it will be considered in the part focusing on that symptom.

If there is no RCT reporting results on a specific symtpom, we will report a description of the results provided by other studies.

Then, we will synthesises these data in a critical analyse of the reported literature.

1. **Study schedule:**

| Nov 18 Dec 18 Jan 19 Feb 19 Mar 19 | | | | |
| --- | --- | --- | --- | --- |
| Defining and agreeing about the review question and global methodology  Preparing the protocol. | Finishing the protocol.  Registering the protocol in the PROSPERO database.  Data collection and extraction | Data collection and extraction | Data analysis | Writing and submission |

1. Kent JM. SNaRIs, NaSSAs, and NaRIs: new agents for the treatment of depression. Lancet. 2000;355(9207):911-8.

2. Anttila SA, Leinonen EV. A review of the pharmacological and clinical profile of mirtazapine. CNS Drug Rev. 2001;7(3):249-64.

3. Biswas PN, Wilton LV, Shakir SA. The pharmacovigilance of mirtazapine: results of a prescription event monitoring study on 13554 patients in England. J Psychopharmacol. 2003;17(1):121-6.

4. Carvalho AF, Sharma MS, Brunoni AR, Vieta E, Fava GA. The Safety, Tolerability and Risks Associated with the Use of Newer Generation Antidepressant Drugs: A Critical Review of the Literature. Psychother Psychosom. 2016;85(5):270-88.

5. Montgomery SA. Safety of mirtazapine: A review. Int Clin Psychopharm. 1995;10:37-45.

6. Davis MP, Khawam E, Pozuelo L, Lagman R. Management of symptoms associated with advanced cancer: olanzapine and mirtazapine. A World Health Organization project. Expert Rev Anticancer Ther. 2002;2(4):365-76.

7. Zaini S, Guan NC, Sulaiman AH, Zainal NZ, Huri HZ, Shamsudin SH. The Use of Antidepressants for Physical and Psychological Symptoms in Cancer. Curr Drug Targets. 2018;19(12):1431-55.

8. Richardson LA, Jones GW. A review of the reliability and validity of the Edmonton Symptom Assessment System. Curr Oncol. 2009;16(1):55.

9. Theobald DE, Kirsh KL, Holtsclaw E, Donaghy K, Passik SD. An open-label, crossover trial of mirtazapine (15 and 30 mg) in cancer patients with pain and other distressing symptoms. J Pain Symptom Manag. 2002;23(5):442-7.

10. Kim SW, Shin IS, Kim JM, Kim YC, Kim KS, Kim KM, et al. Effectiveness of mirtazapine for nausea and insomnia in cancer patients with depression. Psychiatry Clin Neurosci. 2008;62(1):75-83.

11. Riechelmann RP, Burman D, Tannock IF, Rodin G, Zimmermann C. Phase II trial of mirtazapine for cancer-related cachexia and anorexia. Am J Hosp Palliat Care. 2010;27(2):106-10.

12. Davis MP, Kirkova J, Lagman R, Walsh D, Karafa M. Intolerance to mirtazapine in advanced cancer. J Pain Symptom Manage. 2011;42(3):e4-7.

13. Bramer WM, Rethlefsen ML, Kleijnen J, Franco OH. Optimal database combinations for literature searches in systematic reviews: a prospective exploratory study. Syst Rev. 2017;6(1):245.

14. Bruera E, Kuehn N, Miller MJ, Selmser P, Macmillan K. The Edmonton Symptom Assessment System (ESAS): a simple method for the assessment of palliative care patients. J Palliat Care. 1991;7(2):6-9.

15. Chang VT, Hwang SS, Feuerman M. Validation of the Edmonton Symptom Assessment Scale. Cancer. 2000;88(9):2164-71.

16. Pautex S, Vayne-Bossert P, Bernard M, Beauverd M, Cantin B, Mazzocato C, et al. Validation of the French Version of the Edmonton Symptom Assessment System. J Pain Symptom Manage. 2017;54(5):721-6 e1.

17. Carvajal A, Hribernik N, Duarte E, Sanz-Rubiales A, Centeno C. The Spanish version of the Edmonton Symptom Assessment System-revised (ESAS-r): first psychometric analysis involving patients with advanced cancer. J Pain Symptom Manage. 2013;45(1):129-36.

18. Kwon JH, Nam SH, Koh S, Hong YS, Lee KH, Shin SW, et al. Validation of the Edmonton Symptom Assessment System in Korean patients with cancer. J Pain Symptom Manage. 2013;46(6):947-56.

19. Hannon B, Dyck M, Pope A, Swami N, Banerjee S, Mak E, et al. Modified Edmonton Symptom Assessment System including constipation and sleep: validation in outpatients with cancer. J Pain Symptom Manage. 2015;49(5):945-52.

20. Kim SH, Shin DW, Kim SY, Yang HK, Nam E, Jho HJ, et al. Terminal Versus Advanced Cancer: Do the General Population and Health Care Professionals Share a Common Language? Cancer Res Treat. 2016;48(2):759-67.

21. Hokka M, Kaakinen P, Polkki T. A systematic review: non-pharmacological interventions in treating pain in patients with advanced cancer. J Adv Nurs. 2014;70(9):1954-69.

22. Moher D, Shamseer L, Clarke M, Ghersi D, Liberati A, Petticrew M, et al. Preferred reporting items for systematic review and meta-analysis protocols (PRISMA-P) 2015 statement. Syst Rev. 2015;4:1.

23. Teunissen SCCM, Wesker W, Kruitwagen C, de Haes HCJM, Voest EE, de Graeff A. Symptom Prevalence in Patients with Incurable Cancer: A Systematic Review. Journal of Pain and Symptom Management. 2007 Jul;34(1):94–104.
