# Supplementary material for: What is the evidence for mirtazapine in treating cancer-related symptomatology? A systematic review
Source: Support Care Cancer. 2019 Dec 19;28(4):1597–606. doi: 10.1007/s00520-019-05229-7 (PMC7036072; doi:10.1007/s00520-019-05229-7)
Supplement: Supplementary file 2 — (DOCX 13 kb) [file 520_2019_5229_MOESM2_ESM.docx]

| **Data base** | **Research strategy / Algorithm** |
| --- | --- |
| MEDLINE | ((((mirtazapine) OR ("Antidepressive agents"[Mesh]))) AND (("Neoplasms"[Mesh]) OR ("Palliative Care"[Mesh]) OR ("Hospice Care"[Mesh]))) AND (("nausea"[Mesh]) OR ("weight loss"[Mesh]) OR ("fatigue"[Mesh]) OR (“constipation”[Mesh]) OR ("pain"[Mesh]) OR ("Pain management"[Mesh]) OR ("dyspnea"[Mesh]) OR ("anorexia"[Mesh]) OR ("sleep initiation and maintenance disorders"[Mesh]) OR ("sleep wake disorders"[Mesh]) OR ("anxiety"[Mesh]) OR (“depression”[Mesh]) |
| WEB OF SCIENCE | ("mirtazapine" OR “antidepressant”) AND ("cancer") AND ("nausea" OR "appetite" OR “loss of weight” OR " fatigue" OR "pain" OR “breathlessness” OR "anxiety" OR “sleep” OR “drowsiness” OR “constipation” OR “depression” |
| SCOPUS | ("mirtazapine" OR “antidepressant”) AND ("advanced cancer" OR ("palliative care" AND "cancer") OR ("metastasis" AND "cancer")) AND ("nausea" OR "appetite" OR " fatigue" OR "pain" OR " breathlessness" OR "anxiety" OR “sleep” OR “drowsiness” OR “constipation” OR “depression”) |
| EMBASE | (mirtazapine AND cancer) AND (nausea OR appetite OR fatigue OR pain OR breathlessness OR anxiety OR sleep OR drowsiness OR constipation OR depression) |
| CENTRAL | ("mirtazapine" OR “antidepressant”) AND ("cancer") AND ("nausea" OR "appetite" OR “loss of weight” OR " fatigue" OR "pain" OR “breathlessness” OR "anxiety" OR “sleep” OR “drowsiness” OR “constipation” OR “depression”) |
| Opengrey | “cancer” AND “mirtazapine”  “cancer” AND “antidepressant” |
| Clinical Trials | 1. “cancer”  2. “Mirtazapine” |
| International Clinical Trials Registery Plateform (WHO) | Mirtazapine AND cancer |
