# Supplementary material for: What is the evidence for mirtazapine in treating cancer-related symptomatology? A systematic review
Source: Support Care Cancer. 2019 Dec 19;28(4):1597–606. doi: 10.1007/s00520-019-05229-7 (PMC7036072; doi:10.1007/s00520-019-05229-7)
Supplement: Supplementary file 3 — (DOCX 21 kb) [file 520_2019_5229_MOESM3_ESM.docx]

| **Trial** | **Number of participants (analysed) and follow-up** | **Design** | **Intervention /Mirtazapine’s’ starting dose (SD) and increased doses/ route / duration before end-point** | **Control** | **Outcomes and Measurements** | **Main findings (for the Mirtazapine group)** | **Comments** |
| --- | --- | --- | --- | --- | --- | --- | --- |
| **Kim et al., 2008**  **Korea** | 42  (28 for the outcome nausea)  Follow-up completion rate : 0.4 | Prospective non-randomized open-labelled study | Mirtazapine PO  SD 15mg at bedtime  Escalation up to 45 mg based on clinical judgement.  4 weeks treatment | Non-controlled | Assessments at baseline, day 1, 3, 5, 7, 14 and 28.  **Primary outcomes:**  Nausea and vomiting (CGI in 7 points)  Sleeping disorders (C-LSEQ)  **Secondary outcomes:**  Reduce appetite (MADRS subscale)  Reduced sleep (MADRS subscale)  Depression and anxiety (MADRS and C-LSEQ)  Pain (SF – 36)  Quality of life (EQ-5D)  **Safety:**  Side-effects (UKU) | CGI for nausea improved of 2.0 (p<0.001) after day1. Changes in patients undergoing chemotherapy were significantly greater (p<0.001).  C-LSEQ amount of sleep improved of 3.2h (p<0.001), the ease of getting to sleep improved of 3.2 points (p<0.001), the quality of sleep improved of 1.8 points (p< 0.001) and ease of waking in the morning of 0.7 points (p<0.001).  MADRS for reduce appetite decreased of 1.3 (p<0.001)  Ease of getting to sleep and waking in the morning also significantly improved (p< 0.001) and reduced sleep (MADRS) significantly decreased.  C-LSEQ on anxiety and depression decreased of 0.5 (p<0.01)  Pain on SF-36 decreased of 1.1 (p<0.01)  The five items of the EQ-5D improved significantly at every assessment (p<0.01 at end-point) | Effectiveness of the treatment starts from day 1 for nausea and sleeping disorders.  Sedation was exacerbated in 36% but decreased to 8% on day 7 and 0% on day 14.  Dizziness was exacerbated in 14% but it gradually decreased and 0% experienced and exacerbation of dizziness at day14.  Suggests an improvement of the health-related quality of life. |
| **Cankurtaran et al., 2008 Turkey** | 53  Follow-up completion rate : 0.66 | Prospective randomized controlled trial  Requalified as a prospective non-randomized open-labelled study | Mirtazapine PO plus supportive therapy  SD and management are unknown  6 weeks treatment | Supportive therapy (patients who disagreed to take psychotropic drugs)  Or  Imipramine plus supportive therapy | Assessments at baseline, week 3 and week 6.  **Outcomes:**  Nausea (single symptom scale)  Vomiting (single symptom scale)  Reduce appetite (single symptom scale)  Weight (kg)  Sleep disorders (HAM-D)  Depression(HADS-D)  Anxiety (HADS-A)  Pain (single symptom scale) | Single symptom scale rating was not significantly improved for nausea ( p=0.28)  No significant difference has been found in anti-emetic drugs use.  ingle symptom scale ratting was not significantly improved for appetite (p=0.81)  Weight was not significantly improved (p=0.47).  Sleep disorders were significantly improved for early , middle and late insomnia (p=0.001 for each)  HADS-D decreased of 4.7 (p=0.003)  HADS-A decreased of 3.7 (p=0.025)  Single symptom scale rating for pain was not significantly improved (p=0.3) | The study used non-validated tools to assess pain, nausea, vomiting and reduced appetite.  Significant improvement in the sleep quality, depressive symptoms and anxiety.  Does not improved nausea, appetite, or weight.  No statistical analysis are available comparing the arms.  The dosages of mirtazapine and amitriptyline are lower than the recommended ones and escalating doses was unclear. |
| **Riechelmann et al., 2010, Canada** | 17  Follow-up completion rate : 0.29 | Prospective non randomised experimental study | Mirtazapine PO  SD 15mg at bedtime for 3 days before escalation to 30mg if needed.  8 weeks treatment | Non-controlled | Assessments at baseline, week 2, 4 and 8.  **Primary outcome:**  Weight ( proportion of patients gaining at least 1kg after 4 weeks)  **Secondary outcomes:**  Appetite (ESAS items)  Health-related quality of life (FAACT)  **Other reported outcomes:**  Fatigue, pain, nausea, depression, anxiety, drowsiness, breathlessness (ESAS items) | For weight, at week 4 and 8, 24% and 17% of patients respectively gained weight  For appetite, at week 4 and 8, 24% and 24% of patients respectively improved their appetite  No other symptoms assessed by the ESAS were found to be statistically significant.  Health-related quality of life improved for 12% of the patients. | No statistical analysis is available.  Mirtazapine improved weight and appetite.  One patients withdrew because of mild drowsiness, confusion and blurred vision and one because of dry-mouth and drowsiness.  Patients with moderate to severe depressive symptoms were excluded. |
| **Ozsoy et al., 2015 Turkey** | 60  Follow-up completion rate : 1 | Prospective non-randomised experimental study | Mirtazapine PO  SD from 15-30mg at bedtime  Approximatively 6 weeks | Patients who were not diagnosed as depressive (no placebo) | Assessments at baseline and after approximately 6 weeks of treatment.  **Primary outcome:**  Weight (kg and BMI)  Ghrelin and leptin levels  **Secondary outcomes:**  Depression (HAM-D)  Anxiety (HAM-A) | Weight significantly decreased in the Mirtazapine group from baseline to week 6 of 5.27 kg (t=6.299, p<0.001).  Ghrelin level did not changed significantly from baseline to week 6 (p=0.064).  Leptin level did not changed significantly from baseline to week 6 (p=0.167).  BMI significantly decreased in the Mirtazapine group from baseline to week 6 of 1.88 (t=6.240, p<0.01). | Patients and controls differs in terms of weight, BMI and depressive disorders before starting the study.  The Mirtazapine did not mitigated the radiotherapy-induced cachexia and anorexia. |
| **Ersoy et al., 2008 Turkey** | 19  Follow-up completion rate: 0.9 | Prospective non-randomized experimental study | Mirtazapine PO  SD from 15mg at bedtime for 4 weeks before escalation to 30mg in the absence of significant response.  6 month treatment | Non-controlled | Assessment at baseline, end of week 4, 12 and 24.  **Primary outcome:**  Anxiety, sleep disorders (HAM-D specific items, positive treatment defined as a reduction over 50% from baseline)  **Secondary outcomes:**  Depression (HAM-D)  Safety (blood pressure, routine hematologic and biochemistry tests) | All of the patients obtained at least an improvement of 50% of their initial HAM-D scores.  HAM-D anxiety index significantly decreased of 3.0 points in 3 month and 3.2 points in 6 month (compared to baseline)  HAM-D sleep index decreased of 3.7 in 6 month.  HAM-D score decreased of 18.8 in 6 month (p<0.001)  HAM-D depression index decreased of 7.48 (p<0.001) | Mirtazapine significantly reduced the HAM-D ratings at week 24.  Two patients dropped out for unclear reasons but not adverse event.  One patient had intentional tremor, 2 had fatigue, 2 gained weight and 1 had restless legs |
| **Theobald et al., 2002 US** | 20  Follow-up completion rate : 0.55 | Open-labelled cross-over trial | Mirtazapine PO  SD from 15mg or 30 mg with a cross-over 3 weeks later  6 weeks treatment | Cross-over trial | Assessments at baseline and week 1, 4 and 7.  **Primary outcomes:**  Nausea at its worst (NRS)  Appetite at its worst (NRS and ZSDS specific item)  Depression (ZSDS, MPAC)  Pain (MPAC)  Health-related quality of life (FACT-G) | Nausea was not significantly improved  Mean NRS evaluation was not significantly improved for appetite but the ZSDS item for appetite significantly improved ( F=6.9, p<0.05)  Depression (overall ZSDS score) was improved significantly at week 7 (F=8.2, p<0.05) independently of the dosage, no statistical difference was found using the MPAC.  Pain intensity or pain relief were not significantly different.  FACT-G score significantly improved (F=5.7, p<0.05) | No statistical difference has been found between 15mg and 30 mg daily in alleviating pain, nausea, anorexia,  Ratings of scales measuring depression showed a significant improvement.  Suggests an improvement of the health-related quality of life.  Seven dropped out because they died or became too ill to take the treatment, 6 were hospitalized in another clinic, 2 were lost to follow-up and one has his treatment stopped by his GP for unclear reason.  No adverse event is reported. |
| **Davis et al., 2011 US** | 23  Follow-up completion rate : 0.40 | Letter to the editor reporting a prospective non-randomized experimental study | Mirtazapine PO  SD was 15mg for a week before escalation to 30mg if no improvement.  15 days treatment | Non-controlled | Assessments at baseline, day 1, 8 and 15.  **Primary outcome:**  Quality of life ( EORTC-QLQ-C30)  **Secondary outcomes:**  Nausea, anxiety and insomnia (EORTC-QLQ-C30)  **Safety:**  Toxicity (CTCAE) | The quality of life improved in 9% from week 1.  Nausea response rate was 38%.  Insomnia response rate was 44%  Anxiety response rate was 38% | No statistical analysis available.  Very high attrition risk due to a loss of follow-up.  Authors assumes that the drug-tolerance in advanced cancer patients is different than in depressive patients.  Three patients experienced grade 3 toxicity and 1 grade 4 toxicity (16% dropped out because of toxicity graded by a physician)  42% of participants dropped out because of side-effects (9 for somnolence, 3 for delirium or hallucinations, 3 for xerostomia, 2 for nausea, 2 for fatigue, 1 for insomnia). |
| **Van Gool, 2003 Netherland** | 20  Follow-up completion rate : 0.66 | Letter to the editor reporting a prospective non-randomised experimental study | Mirtazapine PO combined with psychotherapy.  SD was 30mg daily, escalation to 45 or 60mg was possible if needed  8 weeks treatment. | Non-controlled | Assessment at baseline, week 2 and 8.  **Primary outcome :**  Depression (CGI) | Authors argue that CGI improved for depression but no statistical analysis are provided | Ten patients dropped out, mostly because of sedation |
| **Nishihara et al., 2013 Japan** | 25  Follow-up completion rate : 0.68 | Prospective randomized controlled trial | Mirtazapine PO plus Pregabalin 25mg every 8h  SD was 7.5mg twice a day  14 days treatment | Pregabalin 50mg every 8h  Or  Pregabalin 25mg every 8h plus imipramine 5mg every 12h | Assessments at baseline, between days 1 and 7, and between days 10 and 14.  **Primary outcome:**  Pain (NRS for the 24h average intensity and paroxysmal pain and daily opioid doses)  Daily opioid doses | NRS for paroxysmal and average pain were significantly lower in the group with mirtazapine from baseline to end-point (p<0.05).  No difference has been found for the daily doses of opioids. | Patients’ selection criteria included intractable pain related to bone metastasis.  The pregabalin group has been cancelled due to uncontrolled pain.  A few patients developed mild dizziness and drowsiness in all 3 arms. |
| **Cao et al., 2018 China** | 95  Follow-up completion rate : unclear | Prospective randomized controlled study | Mirtazapine PO plus aprepitan, a 5HT3 receptor antagonist and dexamethasone 7.5mg  SD was 15mg on day 2-4 of chemotherapy cycles.  2 days treatment. | Aprepitan, a 5HT3 receptor antagonist and dexamethasone 7.5 mg | Assessments at baseline, hour 24, hour 120  **Primary outcomes:**  Vomiting (complete response to vomiting in the delayed phase from 25 to 120h after starting the chemotherapy, complete response during acute phase from 0 to 24h after chemotherapy, complete response in the overall period from 0 to 120h after chemotherapy and complete control during the three period above) | Delayed emesis complete response rate was significantly increased during the first cycle (78.3% vs 49.0%, p=0.003) and third cycle (88.2% vs 55.0%, p = 0.010)  Overall emesis complete response rate was significantly increased (  Delayed and overall complete response rates were significantly improved in the first (70.0% vs 45.7%, p=0.049), second (50.0% vs 25.7%, p=0.043) and third cycle (88.2% vs 50.0%, p=0.010) cycles. | Mirtazapine is efficient in addition to other anti-emetic treatments in alleviating chemotherapy-induced emesis.  Adverse effect reported in the mirtazapine group were Somnolence and weight gain |
| **Raddin et al., 2014 US** | 18  Follow-up completion rate : 0.86 | Prospective non-randomized open-labelled study | Mirtazapine PO  SD was 7.5mg daily, escalation to 15mg or 30mg was possible based on clinical assessment.  9 weeks treatment. | Citalopram 10mg daily, escalation to 20mg and 40mg was possible if needed | Assessments at baseline, week 1, 2,3,4,6 and 9.  **Primary outcomes:**  Weight (kg)  Depression (PHQ-9)  **Secondary outcomes (only assessed at baseline, week3,6 and 9):**  Sleep disorders (PSQI)  Fatigue (FACT-Fatigue)  Health-related quality of life (FACT-G) | Weight did not significantly improved.  Mirtazapine did not improved the PHQ-9 in the overall population.  Excluding active dying patients PHQ-9 score improved of 7.6points (95% CI [2.9-12.2] in 9 weeks.  Sleep disorders did not significantly changed overall or in the amount of hours of sleep.  Fatigue did not significantly changed.  Health related quality of life significantly improved in non-dying patients of 19.1 points on the FACT-G after 9 weeks of treatments. The difference was not statistically significant in the overall sample. | Medications have been chosen based on clinical experience.  Mirtazapine seems to be especially relevant for non-actively dying patients.  3 patients dropped out in the mirtazapine group, two because of nightmares and one because of an alteration of his clinical status.  Other adverse events (all arms together) were fatigue, nausea, decreased appetite, diarrhoea, insomnia and dry mouth). |
| **Kumar et al., 2017 India** | 28  Follow-up completion rate: unclear | Prospective non-randomized study reported in an abstract | Mirtazapine PO  SD was 7.5mg daily without any escalation planned.  2 weeks. | Non-controlled | Assessments at baseline and week 2.  **Primary outcome:**  Gastric emptying using radiolabelled solid meal.  **Secondary outcome:**  Anorexia (reported as no anorexia, mild anorexia, moderate anorexia or severe anorexia) | At baseline 13.8% of particpants had gastric emptying over 75 % whereas they were 31.0% at end-point. Mean percentage of gastric emptying increased from 55.0±21.7% to 64.5±21.4%.  Anorexia was reported at baseline as mild in 10.3%, moderate in 41.4% and severe in 48.3% of the participants. At end-point they were 23.3% to report a complete alleviation of anorexia, 62.1% to suffer mild anorexia and 13.8% to suffer moderate anorexia. No participant reported severe anorexia. | The report of the results is too unclear to properly assess the risk of bias.  No comparative statistical analysis have been performed to compare results about the outcome anorexia. |
| C-LSEQ : Chonnam National Hospital-Leeds Sleep Evaluation Questionnaire  CGI: Clinical Global Impression scale  EQ-5D: EuroQoL(EQ)-5D  ESAS : Edmonton Symptom Assessment Scale  HAD or HAM-D : Hamilton Depression Scale  HADS: Hospital Anxiety and Depression Scale  FAACT: Functional assessment of anorexia/Cachexia Therapy  FACT-G: Functional assessment of cancer Therapy - General | | | | | NRS: numeric rating scale  PHQ-9 : Patient Health Questionnaire 9  SD: starting dose  UKU: UKU side-effect rating scale  VRS: Verbal rating scale  ZSDS: Zung Self-rating Depression Scale  MADRS:Montgomery–Åsberg Depression Rating Scale  MPAC: Memorial Pain Assessment Card | | |

**Supplementary material 3:** Characteristics of included studies
